# Radiation Necrosis, Pseudoprogression, Pseudoresponse, and Tumor Recurrence: Imaging Challenges for the Evaluation of Treated Gliomas

**DOI:** 10.1155/2018/6828396

**Published:** 2018-12-02

**Authors:** Anastasia Zikou, Chrissa Sioka, George A. Alexiou, Andreas Fotopoulos, Spyridon Voulgaris, Maria I. Argyropoulou

**Affiliations:** ^1^Department of Radiology, University Hospital of Ioannina, Ioannina, Greece; ^2^Department of Nuclear Medicine, University Hospital of Ioannina, Ioannina, Greece; ^3^Department of Neurosurgery, University Hospital of Ioannina, Ioannina, Greece

## Abstract

Glioblastoma (GBM) is the most common primary malignant type of brain neoplasm in adults and carries a dismal prognosis. The current standard of care for GBM is surgical excision followed by radiation therapy (RT) with concurrent and adjuvant temozolomide-based chemotherapy (TMZ) by six additional cycles. In addition, antiangiogenic therapy with an antivascular endothelial growth factor (VEGF) agent has been used for recurrent glioblastoma. Over the last years, new posttreatment entities such as pseudoprogression and pseudoresponse have been recognized, apart from radiation necrosis. This review article focuses on the role of different imaging techniques such as conventional magnetic resonance imaging (MRI), diffusion-weighted imaging (DWI), diffusion tensor imaging (DTI), dynamic contrast enhancement (DCE-MRI) and dynamic susceptibility contrast (DSE-MRI) perfusion, magnetic resonance spectroscopy (MRS), and PET/SPECT in differentiation of such treatment-related changes from tumor recurrence.

## 1. Introduction

Glioblastoma (GBM) is the most malignant primary brain tumor in adults with dismal prognosis [1]. Treatment involves gross total excision, when possible, followed by radiotherapy with concurrent and adjuvant temozolomide-based chemotherapy. Nevertheless, nearly all tumors recur. During follow-up, radiotherapy and chemotherapy may produce new lesions that may mimic tumor progression or recurrence on imaging. Accurate detection of glioma recurrence is of paramount importance as it can change the patient's management [2, 3]. To date, several imaging modalities have been employed for the differentiation of glioma recurrence from treatment-induced changes [2–5]. Herewith, we reviewed the current evidence on the ability of imaging techniques such as MRI, SPECT, and PET for the detection of glioma recurrence/progression.

### 1.1. Radiation Necrosis

Radiation necrosis (RN) in patients with malignant gliomas is a severe local tissue reaction to radiotherapy. It generally occurs 3–12 months after radiotherapy but can occur up to several years and even decades later [2, 6]. A complete understanding of the pathophysiology of RN and chemotherapy-induced injury to the central nervous system is useful in understanding and interpreting the conventional and advanced diagnostic imaging findings. The proposed mechanisms by which radiation-associated neurotoxicity may be developed are vascular injury, glial and white matter damage, and the impact on the fibrinolytic enzyme system [7]. In acute radiation-induced injury, transient vasodilatation occurs with variable changes in capillary permeability that sometimes manifest as vasogenic edema. In chronic radiation-induced injury, vascular endothelial damage takes place. Animal studies showed that, in the beginning, there were vascular abnormalities and parenchymal changes in the brain followed. The pathological findings are mainly endothelial damage, vascular dilation, and telangiectasia. These have an effect on capillary permeability that produces cytotoxic and vasogenic edema [8]. Thus, vascular damage has a critical role in the development of radiation-induced effects in the brain; however, further research is needed. One different concept is that cytokine release may promote angiogenesis, which is associated with capillary leakage. The main cytokine secreted after irradiation is tumor necrosis factor-alpha (TNF-*α*). TNF-*α* upregulates other cytokines that induce apoptosis of the endothelial cells, astrocyte activation, and blood-brain barrier (BBB) permeability [9, 10]. The vascular endothelial growth factor (VEGF) induces small vessel permeability and causes cerebral edema. Increased VEGF expression has been reported in the white matter after radiotherapy. VEGF expression has been associated with the magnitude of edema and breakdown of the BBB. Presence of radiation necrosis has been linked to increased VEGF expression [11].

Glial and white matter damage happens after irradiation. Although neurons are relatively insensitive to radiation, it has been shown that oligodendrocytes have higher sensitivity to ionizing radiation and their damage is associated with radiologic evidence of the demyelination that follows [12]. Additionally, there are effects from radiation on the fibrinolytic enzyme system such as the tissue plasminogen and urokinase plasminogen activators, which produce several effects on blood vessels and brain tissue. Sawaya et al. examined irradiated rat cervical spinal cords and proposed the role of plasminogen activators in the pathogenic pathways of radiation damage. An absence of tissue plasminogen activator and an increase in urokinase plasminogen activator leading to cytotoxic edema and tissue necrosis have been reported [13]. Based on that, efforts have been made in the development of radiosensitizers, agents that can be administered prior or concurrent with radiotherapy in order to augment the sensitivity of the tumor, while not affecting normal brain tissue. Thus, radiation dose reduction can be made [14]. In glioblastoma treatment, the chemotherapeutic agent temozolomide is an example of a radiosensitizer. When standard radiotherapy was compared with identical radiotherapy with concomitant temozolomide, an increase in the median survival was found (12.1 vs 14.6 months) [15]. A better understanding of the molecular pathways involved in tumor's radioresistance may reveal new potential therapeutic targets [16, 17].

Radiation necrosis is characterized histopathologically by fibrinoid necrosis of blood vessel walls, with adjacent perivascular parenchymal coagulative necrosis. Collections of abnormally dilated and thin-walled telangiectasias can be also observed. Hyalinization caused vessel wall thickening and is a late finding. Focal and diffuse demyelination constitutes the white matter changes observed.

### 1.2. Pseudoprogression

After completion of RT, with or without concomitant TMZ, patients with high-grade brain tumors can present with new lesions or with an increase in contrast-enhancing previous lesions and perilesional edema. Pseudoprogression has been reported to occur predominantly (in almost 60% of cases) within the first 3 months after completing treatment, but it may occur later after treatment with iomustine and temozolomide [18, 19]. The O^6^-methylguanine-DNA methyltransferase (MGMT) methylation tumor's status has been associated with pseudoprogression occurrence and 2/3 of MGMT methylated tumors exhibit pseudoprogression, 11% early progression, and 25% stable disease. Brades et al. proposed that tumors with methylation of the MGMT promoter, due to greater effect of the combination of temozolomide and radiotherapy to residual tumor, produce a temporary worsening of imaging characteristics which are characterized as pseudoprogression [20]. Hegi et al. showed that patients with methylated MGMT promoter status had better median overall survival (43.6 months vs 16.8 months) [21]. On the other hand, a 60% probability of early true tumor progression in unmethylated MGMT promoter tumors was reported [21]. The exact pathophysiological features of pseudoprogression and the associated molecular changes require further research. Pseudoprogression may constitute an overresponse to effective therapy and is associated with damage to the endothelium, BBB disruption, and oligodendroglial injury [18, 22].

It is important for pathologists to be aware of concerns regarding the accurate diagnosis of this phenomenon. Heterogeneity might be the hallmark when analyzing biopsy samples in these cases. Even within pathologists, there might be variability in the interpretation of findings [23].

### 1.3. Pseudoresponse

Recently, anti-VEGF agents have been utilized for high-grade glioma treatment in several trials. Anti-VEGF agents produce “normalization” of the blood-brain barrier, sometimes within hours. On imaging, there is a reduction in the degree of enhancement by the tumor and a decrease in the surrounding edema on fluid-attenuated inversion recovery (FLAIR). Such an imaging appearance, which imitates a favorable treatment response, is termed “pseudoresponse” because this is due to alterations in vascular permeability instead of tumor response to treatment. So, this radiologic response should be interpreted with caution. Furthermore, studies have shown that antiangiogenic agents, although they cause great imaging changes of the tumor's appearance, overall survival has only a modest increase. Additionally, there might be a rebound effect later with the presence of enhancement and edema [24, 25].

## 2. Diagnostic Imaging Modalities

### 2.1. Conventional and Advanced MR Imaging Techniques

Imaging plays a key role in assessment of response to various treatment regimens for high-grade gliomas. T1-weighted contrast-enhanced MR imaging should be used within the 2 days after surgery in order to assess extent of resection and no later than 72 hours after operation. Moreover, increased contrast enhancement detected by MR imaging just after or during treatment can be produced by several causes such postoperative changes, microischemic lesions, and treatment-associated inflammation. For years, a set of guidelines by Macdonald et al. (Macdonald criteria) were used to evaluate the tumor's treatment response. Based on that, four response categories were identified: complete response, partial response, progressive disease, and stable disease [26]. Limitation of this scheme was that the presence of post-treatment contrast enhancement might not be associated with tumor activity but with BBB disruption. Furthermore, based on the latest RANO criteria, the nonenhancing tumor's component should also be evaluated for decision-making [27]. The most common imaging patterns of radiation necrosis in conventional MRI include a single lesion arising at the resection cavity which can be interpreted for recurrent tumor; a necrosis far from the primary tumor site may mimic multifocal glioma; the “Swiss cheese” pattern can be visualized as diffuse enhancements at the margins between the cortex and white matter.

Regarding the advanced MR techniques, a recent meta-analysis that included 35 studies of all diagnostic MRI techniques in high-grade glioma patients after treatment showed that advanced MRI techniques had higher diagnostic accuracy than conventional MRI for the detection of tumor progression. MR spectroscopy showed the highest diagnostic accuracy followed by perfusion MRI. The sensitivity and specificity of MRS were 91% and 95%, respectively [28]. In a retrospective study of 15 patients with lesions suspicious for glioma progression, a cutoff value of 1.30 in ADC ratio, 2.10 for rCBV ratio, 1.29 for Cho/Cr ratio, and 1.06 for Cho/NAA ratio had a diagnostic accuracy of 86.7%, 86.7%, and 84.6%, respectively. When an analysis of a combination of parameters was performed, the diagnostic accuracy reached 93.3% [29]. Patel et al. also performed a systematic review and meta-analysis to evaluate whether DSC and DCE metrics could differentiate recurrent glioma from posttreatment changes, including both pseudoprogression and radiation necrosis. The meta-analysis included 28 studies, 13 of which evaluated pseudoprogression. The results showed that, for DSC, the pooled sensitivity was 90% and the specificity was 88%. For DCE, the pooled sensitivity was 89% and the specificity was 85% [30]. It is of notice that a recent survey on glioma imaging practices of the members of the European Society of Neuroradiology from 220 institutions showed that perfusion MRI is widely used (85.5%). Spectroscopy is used mainly for specific indications [31].

### 2.2. PET

In a study of fifty consecutive patients, hybrid ^11^C-methyl-L-methionine (^11^C-MET) PET/MRI showed significant higher accuracy than MRI (96% vs 82%) and higher to ^11^C-MET PET (96% vs 88%) to differentiate treatment-related changes from true progression in recurrent glioma, based on RANO criteria [32]. A maximum tumor-to-brain ratio (TBR) of 1.83 and mean TBR of 1.5 were found as optimal cutoff values for discriminating between these two entities [32]. ^11^C-METPET/CT differentiated recurrence from no recurrence with a cutoff max T/N ratio of 1.9, with a sensitivity of 94.7% and a specificity of 88.89% [33]. A limitation of ^11^C-MET is the need for an on-site cyclotron due to short half life of ^11^C, making widespread use of this tracer difficult [34].

O-(2-(18)F-fluoroethyl)-L-tyrosine (^18^F-FET) is an artificial amino acid and a promising tracer for the detection of recurrent glioma. ^18^F-fluorine tracers do not require an on-site cyclotron. In a study of 22 glioblastoma patients that presented with increased enhancement of lesions or new contrast-enhancing lesions within the first 3 months after completion of radiochemotherapy, ^18^F-FET PET could identify pseudoprogression with 96% accuracy. Moreover, survival analysis showed that a max T/N ratio lower than 2.3 predicted a significantly longer overall survival [35]. Compared to conventional MRI, ^18^F-FET PET had higher accuracy (93% vs 86%, respectively) [30]. Using dynamic and static ^18^F-FET uptake parameters, late pseudoprogression could be differentiated from glioblastoma recurrence, with a cutoff value of max T/N of 1.9 with 84% sensitivity and 86% specificity. Concerning clinical decision-making, all cases with max T/N ratio greater than 2.4 were glioblastoma recurrence and all cases with max T/N ratio lower than 1 were pseudoprogression [36]. In a well-designed prospective study that compared ^11^C-MET PET to ^18^F-FET, both tracers showed the same sensitivity (91%) and specificity (100%) for differentiating tumor tissue from treatment-related changes [38].

The most widely available PET tracer is the 2-deoxy-2-(18F) fluoro-D-glucose (^18^FDG), whose function is based on glycolytic metabolism. Thus, lesions with high glucose metabolism show increased uptake. One major disadvantage of ^18^F-FDG is the high uptake in the normal brain; thus, a lesion is not readily identifiable at all times [3]. The accuracy of ^18^F-FDG PET in the detection of recurrent tumor has been questioned, and reported sensitivities and specificities vary widely [39]. However, a recent meta-analysis showed that ^11^C-MET does not have noticeable advantage over ^18^F-FDG [40]. The use of ^11^C-choline PET/CT demonstrated higher sensitivity and specificity compared with ^18^F-FDG PET/CT for distinguishing recurrent brain tumor from radionecrosis [41]. Similarly, another promising PET radiopharmaceutical represents the 3,4-dihydroxy-6-[(18)F] fluoro-phenylalanine (^18^F-FDOPA), which may be highly sensitive and specific for detection of recurrence in glioma patients [42]. Jena et al. studied 35 glioma-treated patients harbouring 41 enhancing lesions with ^18^F-FDG PET/MRI imaging. The accuracy of perfusion MRI (rCBV) for detecting glioma recurrence was 77.5%, 78% for ADC mean, 90.9% for Cho/Cr, 87.8% for max T/N, and 87.8% for mean T/N. On multivariate ROC analysis, the maximum area under the curve (AUC) of 0.935 ± 0.046 was achieved when ADC mean, Cho/Cr, and TBRmean were combined [43].  Table 1 summarizes representative PET studies for the detection of recurrent glioma [32–34, 36, 37, 43–47].

Other PET radiopharmaceuticals that are under investigation to differentiate between progression from pseudoprogression or tumor recurrence include ^18^F-fluoromisonidazole (FMISO) PET/MR [48], 4-borono-2-[(18)F]-fluoro-phenylalanine (^18^F-FBPA) PET [49], [(18)F]-fluoromethyl-dimethyl-2-hydroxyethylammonium (^18^F-fluoromethylcholine) PET [50], and 3′-deoxy-3′-(18)F-fluorothymidine (FLT) PET [51].

### 2.3. SPECT

Compared to PET, SPECT is widely available and of lower cost; however, it has lower spatial resolution. Several SPECT tracers have been evaluated mainly for the differentiation of glioma recurrence from radiation necrosis. ^201^Thallium (^201^Tl) was one of the first SPECT tracers studied [52]. Given that there is no Tl uptake in the healthy brain, tumor recurrence can be readily identified. The sensitivity of ^201^Tl SPECT for the detection of recurrent glioma ranged from 0.43 to 1.00, and the specificity ranged from 0.25 to 1.00 [52]. Technetium-99m-labeled compounds are superior to ^201^Tl given the 140 keV *γ*-ray energy and high photon flux that correspond to higher spatial resolution and less radiation burden to the patient [53]. ^99m^Tc-sestamibi and ^99m^Tc-tetrofosmin have been found suitable for the detection of recurrent tumors. Both tracers have no uptake in the healthy brain except areas with absence of BBB such as choroid plexus. A cutoff value of lesion-to-tumoral uptake ratio around 4 has been reported for the differentiation of recurrent tumor from radiation necrosis [53–55].

It is of interest that ^99m^Tc-tetrofosmin SPECT was found to have the same accuracy with perfusion MRI to detect recurrent tumor following glioma treatment [55]. ^99m^Tc-methionine andiodine-123-a-methyl tyrosine (^123^I–IMT) has been also evaluated for the detection of radiation necrosis [56]. Amino acid-based tracers have an uptake in the healthy brain, contrary to the previous reported tracers. Recently, in a study of 44 patients with suspected glioma recurrence, ^99m^Tc-methionine SPECT/CT had similar diagnostic values with FDG PET/CT and higher than contrast-enhanced MRI for the detection of glioma recurrence. The sensitivity and specificity of ^99m^Tc-methionine, FDG PET/CT, and contrast-enhanced MRI was 75.9% and 90%, 82.8% and 80%, and 87.1% and 30%, respectively [57]. In a meta-analysis that evaluated the diagnostic ability of SPECT in differentiating glioma recurrence from radiation necrosis, the pooled sensitivity was 89% and the specificity 88% [58]. Other promising tracers for SPECT to differentiate tumor recurrence from radiation necrosis include pentavalent technetium-99m dimercaptosuccinic acid (Tc-99m (V) DMSA) [59], bis-methionine-DTPA (Tc MDM) [60], and 99m-technetium glucoheptonate (^99m^Tc-GHA) [61].

## 3. Conclusions

Pseudoprogression is a major concern in glioma-treated patients and has a reported incidence between 10 and 30%. It usually appears several weeks up to approximately 4 months after radiotherapy. Thus, patients are not eligible to enter clinical trials within 4 months after treatment, because of the risk of unreliable results. Contrary to radiation necrosis, the pathophysiology of pseudoprogression remains elusive and further studies are needed. To date, no single technique provides a reliable detection of glioma recurrence. An imaging modality that could reliably detect tumor progression would be of paramount importance. One major limitation in all previously reported studies is the absence of histopathological confirmation of the final diagnosis in all cases. This might be due to lesion's location in an eloquent brain region or patient denial for biopsy or further surgery. Given all the above, the main focus of future studies should be on the discrimination of recurrence from no recurrence. Well-designed future studies combining several imaging modalities should be performed.

## Figures and Tables

**Table 1:** Cutoff values of PET tracers for the detection of glioma recurrence.

Study	Modality	Tracer	Parameter	Cutoff	Sensitivity (%)	Specificity (%)
Deuschl et al. [32]	PET/MRI	^11^C-methionine	Max T/N	1.8	97.14	93.33
			Mean T/N	1.3		
Tripathi et al. [33]	PET/CT	^11^C-methionine	Max T/N	1.9	95	89
Terakawa et al. [34]	PET	^11^C-methionine	Mean T/N	1.58	75	75
Herholz et al. [44]	PET	^11^C-methionine	T/N	1.47	76	87
Garcia et al. [45]	PET	^11^C-methionine	T/N	2.35	90.5	100
Takenaka et al. [46]	PET	^11^C-methionine	T/N	2.51	91.2	81.4
Galldinks et al. [36]	PET	^18^F-FET	Max T/N	2.3	100	91
Kebir et al. [37]	PET	^18^F-FET	Max T/N	1.9	84	86
Jena et al. [43]	PET/MRI	^18^F-FDG	Max T/N	1.579	93.3	72.7
			Mean T/N	1.179	90	81.8
Enslow et al. [47]	PET	^18^F-FDG	T/N	1.83	100	75
			Max T/N	6.2	90.9	75
